# Letter from Paris

**Published:** 1890-04

**Authors:** P. Dubois

**Affiliations:** President of the Odontological Society of Paris


### Foreign Correspondence.

---

LETTER FROM PARIS.

To the Editor:

Although it is rather late to speak to the readers of the International Dental Journal of the International Dental Congress of Paris, yet it may not be without interest to them to learn in brief of the most important communications received.

In the section of anatomy and physiology, normal and pathological, I would mention a learned paper by M. Rothmann, of Buda-Pesth, on the "Patho-Histology of the Pulp and Periosteum." He brought to the support of his conclusions a beautiful series of microscopic specimens which give great credit to M. Rothmann as a microscopist.

Permit me also to call your attention to two communications of your correspondent, one, "The Teeth of the French," and the other an "Essay on the Terminology of the Principal Diseases of the Teeth and Mouth." I consider that it would be of great advantage in the study and discussion of obscure points in the science of odontology, if dentists would unite on one common nomenclature. On account of the general familiarity with the pathological anatomy of the mouth and dental organs, it seems to me best to make this the foundation or beginning for our classification. Any of our readers interested in this matter will find it treated at length, and the classification proposed, in the October number of *L'Odontologie*.

In the section of therapeutics the subject of diseased pulps received a long and exhaustive discussion. We are not convinced of the wisdom of filling-root canals immediately on opening. Personally I treat the canals and fill temporarily with gutta-percha, filling permanently after the second, third, and fourth sitting, according to the conditions of the case. By this method I claim to have only from three to five failures in a thousand. Dr. Cunningham's statistics give four cases of periostitis out of forty-five treated.

M. Heide, of Paris, gave his method of filling cavities with inlays cut from natural teeth, and M. Guérini, of Naples, with coral of whitish color.

Cocaine and nitrous oxide are used to a great extent in France, owing somewhat, probably, to legislation, which prohibits general anæsthesia by those who do not have the degree of M.D.

M. Bleischsteiner, of Gratz, always uses the aqueous solution of cocaine, first sterilizing the water with a weak solution of corrosive sublimate. His method is to inject at several places, but only a little in each place. M. Poinsot, of Paris, advises a solution in oil of vaseline, medicinal,[1] or oil.

His formula is,—

| | | |
|---|---|---|
| ℞ Pure cocaine, | 5 | centigrammes; |
| Liquid vaseline, | 50 | " |
| Pea-nut (or pistachio) oil, | 50 | " |

This solution is not toxic. I have given the reason in the "Aid to Memory of the Surgeon Dentist." The aqueous solution is quickly absorbed by the blood-vessels and carried to the nerve-centres, while the oily solution remains longer where it was injected and has more of a local effect. During the three years that I have used this formula I have had no accidents whatever, whereas with the aqueous solution I have seen alarming symptoms of syncope, lasting for hours, and in some cases for days. The inconvenience of this preparation is its lack of fluidity and consequent difficulty of injecting it, and more especially if low temperature has caused a recrystallization. For this reason I have modified the formula of M. Poinsot a little. My method is as follows: Into a tube (or phial), of a capacity of two cubic centimetres, put from three to five centigrammes of cocaine (my cocaine is not the hydrochlorate but the pure alkaloid); to the crystals add two drops of chloroform, then equal parts of pea-nut oil and vaseline oil to make a quantity sufficient to fill a hypodermic syringe; that is to say, one gramme or one cubic centimetre. To avoid rapid recrystallization of the cocaine, it is well to warm slightly the phial and also the syringe. Like M. Bleischsteiner, I inject at several points and put the point of the syringe into the tissue but a short distance. By this method there is no fear of accident, and the operation is less dreaded by nervous patients. It is a good idea, after the extraction is completed, to force out, if possible, the injected liquid by pressing the gum hard between the fingers.

In *prosthetic dentistry* I will mention the interesting presentation of M. Michaëls, of Paris. In making metal plates, he treats the mould so that the die will give the plate a slightly wrinkled ap-

---

[1] Not to be confounded with mineral vaseline.

pearance; on the lingual side he flows solder into the depressions (striæ), thus making the plate ridged enough to withstand the strain of mastication.

M. Michaëls made before the members of the Congress a plate having four teeth in forty minutes. The process, I believe, has a great future.

The restoration of the face and maxillæ has been entirely transformed in France, thanks to the good work of M. Martin, of Lyons. The most startling and at the same time the happiest idea of our eminent *confrère* is the *immediate* application of the appliance. The readers of the INTERNATIONAL DENTAL JOURNAL, who are engaged in facial restoration, know how difficult it is to successfully put in position an appliance where there has been resection of the jaw, and the consequent falling away and retraction of the soft tissues left without osseous support. M. Martin's remedy is to make in advance a reproduction in black rubber of the bone to be removed. As soon as the operation is completed, the rubber is fitted into the place where the bone has been removed, and is then held in position by a platinum screw. Over this artificial maxilla the surgeon fastens the flaps of soft tissue with sutures, and the appliance is left in place until the healing is complete. My readers will be astonished, as I myself was, to learn that the false maxilla will be tolerated in contact with so large a wound. The surgeon, M. Olier, who has often employed M. Martin's appliance, makes the following statement: "Is it prudent to disturb, or at least fill, a fresh and irregular wound with a foreign body that may become a permanent source of irritation, and of whose retention there may be doubt? It is remarkable to see how the healthy bone will tolerate the platinum screws used to hold the appliance (hard rubber) in place. It is always, without doubt, a question of antisepsis and the property of heat that are necessary to success. By the manner in which the piece is constructed it is easy to keep it in place. It is sufficient to frequently and methodically irrigate the parts."

It seems to be quite probable that it is on account of the frequent irrigation that the piece is tolerated. M. Martin has made this easy by having the appliance furnished with grooves or canals of irrigation to which a rubber tube can be applied.

I cannot describe here minutely the method of construction and procedure of this class of operations, but will refer the reader to M. Martin's excellent book on the subject.[1] In this book may be found,

[1] "De la Prothése immédiate appliquir à la Risection des Maxillaires." Par C. Martin, Chez Maison, Paris, 1889.

also, other important information in regard to facial restoration, to which your eminent co-laborer, M. Bonwill, and dentists who witnessed the demonstration by M. Martin at the *Exposition Universelle*, will bear testimony.

The clinical sessions were of great interest. M. Bonwill demonstrated his method of gold filling. M. Chauvin transplanted a root on which had been placed an artificial crown. MM. Bleischsteiner, Michaëls, and Rothmann exhibited their work and microscopic slides, which have already been mentioned. M. Cunningham, of Cambridge, showed his diagrams used in teaching at the National Hospital, London. M. Telschow exhibited a dental engine worked by compressed air, also a mallet and saliva-pump attached to the same. He also showed an improved clamp.

MM. Michaëls and Kuhn showed their operating rooms where compressed air is used for the engine and also for running the dynamo.

In regard to the fears expressed by the INTERNATIONAL DENTAL JOURNAL and many other American journals that it was our wish to injure the Congress to be held in Berlin, we have proved that this was not our end. Before adjournment, the Congress gave expression to the wish for another reunion whenever such favorable circumstances should present themselves as was the case with the Congress of Paris.

I hold in grateful remembrance the cordial reception given us by our American *confrères* at Washington. My colleagues of l'École Dentaire, of Paris, and myself have done our best to give a fitting reception to those dentists who have honored us with their presence. We were particularly happy to have had with us our American friends, Bonwill and Harlan, to whom we wish to renew our expressions of regard.

You will see, my dear Editor, that the Congress of Paris has been most useful and happy in its results. It has assisted the progress of dentistry, and strengthened the bond of friendship between the members of the profession throughout the world.

P. DUBOIS,
*President of the Odontological Society of Paris.*

Translated by Dwight M. Clapp, Boston,
Foreign correspondent *L'Odontologie.*

